# Balancing microbial composition through diet

**DOI:** 10.7554/eLife.104560

**Published:** 2024-11-20

**Authors:** L Catalina Acuff, Karen Guillemin

**Affiliations:** 1 Institute of Neuroscience, University of Oregon Eugene United States; 2 Institute of Molecular Biology, University of Oregon Eugene United States; 3 Humans and the Microbiome Program, CIFAR Toronto Canada

**Keywords:** microbiome, protein, enterocytes, *Vibrio cholerae*, kwashiorkor disease, Zebrafish

## Abstract

A reciprocal interaction between gut bacteria and gut cells affects protein absorption in the host.

**Related research article** Childers L, Park J, Wang S, Liu R, Barry R, Watts SA, Rawls JF, Bagnat M. 2024. Protein absorption in the zebrafish gut is regulated by interactions between lysosome rich enterocytes and the microbiome. *eLife* **13**:RP100611. doi: 10.7554/eLife.100611.

The gut of animals is a complex ecosystem consisting of trillions of bacteria and other microorganisms that are unique to every individual. Infants can inherit their first gut bacteria through vaginal birth and breastfeeding, and the microbiome is later reshaped through diet (Ames et al., 2023).

The gut microbiome helps to break down and enhance the absorption of nutrients like carbohydrates and lipids but the role of gut bacteria in protein absorption is less clear. Previous research has shown that in neonatal mammals and fishes, specialized cells lining the gut, called enterocytes, are essential for absorbing protein (Park et al., 2019). Now, in eLife, Michel Bagnat, John Rawls and colleagues from Duke University, University of North Carolina and University of Alabama at Birmingham – including Laura Childers as first author – report how microbes affect protein absorption by enterocytes (Childers et al., 2024).

To follow the fate of proteins in a living organism, Childers et al. used transparent zebrafish larvae to visualize fluorescent proteins in their journey through the gut. The team fed the zebrafish fluorescent protein and isolated all the lysosome-rich enterocytes, or LREs, that took up the glowing cargo, and which had been previously shown to take up protein efficiently. They used various techniques to manipulate zebrafish microbiomes. Fish either had a complete gut microbiome (called conventionally reared) or no gut microbiome (germ-free): germ-free animals were further inoculated with single bacterial strains to generate fish with only one type of bacteria in their gut. These monoassociated fish allowed the researchers to measure precisely how specific bacteria affected protein uptake in the zebrafish gut. They then looked at gene expression in each of these different scenarios.

The results showed that collectively, resident microbes slowed down protein uptake and degradation in LREs. Moreover, the LREs in conventionally reared fish upregulated genes involved in immune responses and bacteria-sensing and downregulated genes linked to the degradation of proteins. To identify the impact of specific bacteria on LREs, Childers et al. tracked the fates of the fluorescent proteins in monoassociated fish and identified a bacterial strain, *Acinetobacter calcoaceticus*, which only slightly reduced protein uptake compared to germ-free fish, while the *Vibrio cholerae* strain significantly decreased how much protein the LREs absorbed.

To explore why the fish colonized by *V. cholerae* absorbed protein so poorly, Childers et al. analyzed the protein uptake machinery of LREs. This revealed that expression of genes encoding the protein uptake machinery in *V. cholerae* monoassociated fish were reduced compared to *A. calcoaceticus-*monoassociated or germ-free animals. Why the expression of these genes changed in the presence of different bacteria remains to be determined. The *V. cholerae* strain used can cause a lot of damage to enterocytes (Ngo et al., 2024). It is possible that LREs down-regulate protein uptake in the presence of harmful bacteria like *V. cholerae* to avoid taking up toxic proteins. Alternatively, the LREs of *V. cholerae*-colonized fish may simply be slightly impaired and less capable of protein uptake. This suggests that individual bacterial strains can influence how much protein gets absorbed by enterocytes.

Childers et al. also explored how the amount of protein remaining in the gut tube affected the composition of the gut microbiome. When they fed zebrafish a low protein diet in comparison to the normal diet, they noted significant shifts in the types of bacteria found in the gut, especially in mutant zebrafish with defects in their protein uptake machinery. Fish fed a low protein diet had less diverse microbiomes.

Understanding what regulates the protein uptake machinery will shed new light on diseases of malnutrition. For example, children with kwashiorkor disease suffer from protein malabsorption (Williams, 2003). Their gut microbiomes appear to play a role in the disease because feces from children with kwashiorkor can cause similar symptoms when introduced into germ-free mice (Smith et al., 2013). Perhaps the gut bacteria of kwashiorkor patients inhibit protein uptake similarly to *V. cholerae* in zebrafish.

Childers et al. show that gut microbes shape the fate of protein in the gut. Future research will explore how specific bacteria affect the protein uptake machinery and how protein availability in the gut affects the microbiome. Already, the findings support the idea that correcting defective microbiomes could be a promising strategy for treating childhood malnutrition (Barratt et al., 2022). The findings also offer a cautionary lesson: dramatically changing the protein content of your diet, for example by consuming protein shakes, could backfire by changing the microbiome, possibly in a way that impairs protein uptake processes in the gut.
